# UvSnx4 is required for conidiation, pathogenicity and stress responses by regulating mitophagy and macroautophagy in *Ustilaginoidea virens*

**DOI:** 10.1007/s44297-023-00002-z

**Published:** 2023-08-10

**Authors:** Huanbin Shi, Shuai Meng, Shuwei Xie, Jiehua Qiu, Nan Jiang, Yanjun Kou

**Affiliations:** State Key Laboratory of Rice Biology and Breeding, China National Rice Research Institute, Hangzhou, 311400 China

**Keywords:** Rice disease, Rice false smut, Virulence, Fungal pathogen, Snx4, Mitophagy, Autophagy

## Abstract

Rice false smut disease, caused by the biotrophic fungus *Ustilaginoidea virens*, threatens rice production and quality. SNX family proteins have a Phox domain in the structure, playing important roles in protein transport and sorting in the endocytic pathway. However, the roles and underlying mechanisms controlled by SNX proteins in development and pathogenicity are still poorly understood in *U. virens*. Here, we characterized the SNX family protein Snx4 in *U. virens*. The transcriptional levels of *UvSNX4* were upregulated at the invasive growth stage. Deletion of *UvSNX4* resulted in defects in mycelial growth, conidiation, pathogenicity, and stress adaptations. Microscopic observation showed that UvSnx4 partially colocalized with mitochondria. Furthermore, UvSnx4 is required for mitophagy and macroautophagy, which are critical for conidiation and pathogenicity in *U. virens*. Moreover, UvSnx4 interacted with the autophagy-related core proteins UvAtg11, UvAtg20, and UvAtg17. In conclusion, our studies suggested that UvSnx4 contributes to development, stress adaptation, and pathogenicity via involvement in mitophagy and autophagy in *U. virens*, which provides insight into the biological functions of SNX proteins in biotrophic filamentous fungi.

**Supplementary Information:**

The online version contains supplementary material available at 10.1007/s44297-023-00002-z.

## Introduction

Rice false smut disease, caused by ascomycetous *Ustilaginoidea virens*, has emerged as one of the most devastating rice diseases in China and other rice-growing countries [1–3]. The typical and only obvious disease symptom of rice false smut is the formation of false smut balls, which are covered with powdery chlamydospores. Rice false smut not only causes serious loss in yield but also compromises food safety due to the synthesis of mycotoxins [4–7]. Under proper humid conditions, chlamydospores germinate to produce hyphae to differentiate secondary spores. *U. virens* infects rice florets at the late booting stage and then forms massive mycelia occupying the seed space to inhibit rice grain filling. In recent years, substantial progress has been made in elucidating the infection mechanisms of *U. virens,* including the disease cycle, functional genomics, and effector-mediated interaction with the host [2]. However, in contrast to other model filamentous fungal pathogens, such as *Magnaporthe oryzae* and *Fusarium graminearum*, the molecular mechanism of *U. virens* pathogenesis remains largely unknown. Understanding the pathogenic mechanism of *U*. *virens* would provide methods and potential targets for the prevention and control of rice false smut disease.

Autophagy is a well-conserved biological process in eukaryotic cells, in which damaged organelles or macromolecules are enclosed by a double-membrane structure of autophagy, autophagosomes, and transported to vacuoles (fungi or plants) or lysosomes (mammals) for degradation and recycling [8–10]. Autophagy can occur under a variety of natural conditions, not only under starvation or stresses but also during cell differentiation or development [11]. In *Saccharomyces cerevisiae*, 43 genes have been identified to be involved in autophagy, and the molecular mechanism of autophagy has been well studied [12–14]. In* U*. *virens*, current knowledge about the underlying regulatory mechanisms of autophagy is very limited, and only the biological roles of three autophagy-related genes, Atg8, Atg14, and Atg7, have been reported [5, 11]. Autophagy is divided into different types according to the selectivity and specialty of degraded substrates, such as nonselective macroautophagy and selective mitophagy. In contrast to well-known macroautophagy, mitophagy is a complex physiological process that can maintain the balance of mitochondrial quality and quantity, keep cellular function properly under unsuitable conditions, and maintain the stability of intracellular environments [15, 16]. In pathogenic fungi, mitophagy has been shown to play important roles in pathogenesis [17–21]. However, thus far, there have been almost no reported data about mitophagy in *U*. *virens*.

In yeast, sorting nexin Snx4 participates in retrograde trafficking of different cargos by interacting with different proteins. Snx4 interacts with sorting nexin Atg20 to form a heterodimeric complex, participating in sorting of the v-SNARE Snc1. The Atg20-Snx4 complex is related to the formation of the phagophore membrane during autophagy. In addition, Snx4 interacts with Snx41, mediating sorting of the integral membrane protein Atg27, which is involved in selective autophagy. Recent studies have shown that Snx4 participates in fine-tuning autophagic activity by contributing to the turnover of autophagy-related gene transcriptional regulators [22]. The roles of Snx4 homologues have also been characterized in other fungi. In the fission yeast *Schizosaccharomyces pombe*, Snx4 forms a homo-oligomer and regulates organelle autophagy by controlling autophagosome size and Atg8 accumulation at phagophore assembly sites. In the rice blast fungus *M. oryzae*, MoAtg24, a homologue of Snx4, is required for conidiation and pathogenicity by mediating mitophagy but not macrautophagy and pexophagy. In addition, in the wheat head blight pathogen *Fusarium graminearum*, FgSnx4 is required for regulating polarized growth and pathogenicity by forming a heterodimer with FgSnx41 and endosomal protein sorting of FgSnc1, a homolog of yeast snc1. Furthermore, FgSnx4 was found to form a complex by interacting with FgAtg1, FgAtg11, FgAtg17, and FgAtg20 to mediate macroautophagy, the cytoplasm to vacuole pathway, and pexophagy. However, the biological roles of UvSnx4 in *U. virens* are unknown.

In this study, we revealed the involvement of *UvSNX4* in the growth, conidiation, stress adaptation, and pathogenicity of *U. virens*. Furthermore, we found that UvSnx4 mediates autophagy and mitophagy, which are necessary for conidiation and pathogenicity in *U. virens*. By testing the interactions between UvSnx4 and autophagy-related proteins, we inferred that UvSnx4 might mediate mitophagy and autophagy by direct interactions with autophagy-related proteins.

## Results

### Identification and deletion of UvSnx4 in *U. virens*

**Fig. 1:**
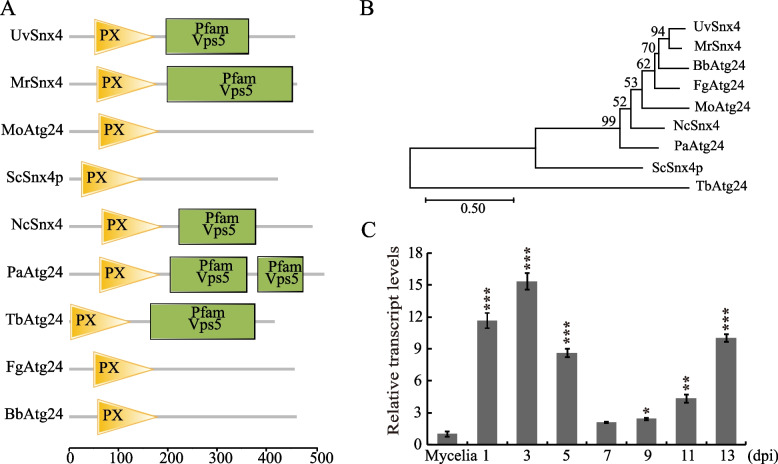
Identification of *UvSNX4* and expression analysis during infection. **A** UvSnx4 contains a PX domain and a Vps5 domain. PX, phosphoinositide binding domain. **B** Phylogeny analysis showed that UvSnx4 is close to the Snx4 homolog in *M. robertsii*. **C** The expression levels of *UvSNX4* are upregulated during infection. Gene expression levels of *UvSNX4* were detected by qPCR with samples at different days post inoculation (dpi) and calibrated to that of mycelia. *UvACTIN* was used as an internal reference gene. The data are presented as the mean ± standard deviation (SD) and were subjected to Student’s *t* test. ***, *P* < 0.001; **, *P* < 0.005; *, *P* < 0.01

To obtain the Snx4 homolog in *U. virens*, the amino acid sequence of ScSnx4 (NP_012498.1) was used as a query for the protein blast search on the NCBI database (https://www.ncbi.nlm.nih.gov/). The gene locus UV8b_01659, showing 55% amino acid identity to ScSnx4, was identified as the homolog of Snx4 and thus named UvSnx4. To further understand the phylogenetic relationship of UvSnx4 with homologues in other fungi, we obtained Snx4 homologues, including *Metarhizium robertsii* Snx4 (XP_007821453.1), *M. oryzae* Atg24 (XP_003716251.1), *S. cerevisiae* Snx4 (NP_012498.1), *Neurospora_crassa* Snx4 (XP_965318.1), *F. graminearum* (XP_011328628.1), *Beauveria bassiana* (XP_008603018.1), *Trypanosoma brucei* (XP_011777027.1), and *Podospora anserina* (CDP22508.1). Sequence identities and similarities were compared by alignment of nine Snx4 homologues (Fig. S1). The results showed that all Snx4 homologues share high sequence identities and similarities. Moreover, functional domain analysis revealed that Snx4 homologues contain a PX domain (phox-homology domain, a phosphoinositide-binding domain) and a Vps5 domain, as predicted by the protein SMART tool (http://smart.embl-heidelberg.de) (Fig. 1A). Phylogenetic analysis showed that UvSnx4 is closest to Snx4 in *M. robertsii* (Fig. 1B). These data indicated that Snx4 is evolutionarily conserved in different fungi.

To investigate the functions of UvSnx4 in the biology of *U. virens*, the gene expression pattern of *UvSNX4* was monitored during the infection stages of *U. virens*. The results of qRT‒PCR analysis showed that the transcription levels of *UvSNX4* were higher at 1, 3, 5, 9, 11, and 13 dpi (days post inoculation) than at the mycelial stage (Fig. 1C). Among them, the expression level at 3 dpi was the highest, which was upregulated 15-fold compared with that of the mycelial stage. The transcriptional expression upregulation of *UvSNX4* at time points postinoculation implicated an important role of UvSnx4 in the infection stage.

To unravel the biological functions of *UvSNX4*, null mutants of *UvSNX4* were constructed by substituting the targeted gene region with the *hygromycin phosphotransferase gene cassette* in the wild-type (WT) strain HWD-2 (Figure S2A). Then, the generated transformants were verified by Southern blot and qRT‒PCR assays. As shown in Figure S2B, the shift from the 2.8 kb band in the WT strain to the 4.4 kb band indicated that the *UvSNX4* gene was successfully deleted in the transformants Δ*Uvsnx4*-15, -18, -36, -69, and -106 (Figure S2B). Two knockout mutants, Δ*Uvsnx4*-15 and -36, were randomly chosen for the following experiments. To explore whether the defective phenotypes observed in the Δ*Uvsnx4* mutants were caused by the disruption of *UvSNX4*, the complementation strain Δ*Uvsnx4*-C was constructed by reintroducing the wild-type copy of *UvSNX4* into the Δ*Uvsnx4*-36 mutant using the ATMT (*Agrobacterium tumefaciens*-mediated transformation) method. Furthermore, the results of PCR and qRT‒PCR assays showed that the transcript abundance of *UvSNX4* in the WT and Δ*Uvsnx4*-C strains reached comparable levels, suggesting that the expression of *UvSNX4* is rescued in the Δ*Uvsnx4*-C strain (Figure S2C and D).

### *UvSNX4* is required for mycelial growth, sporulation, and secondary spore formation

**Fig. 2:**
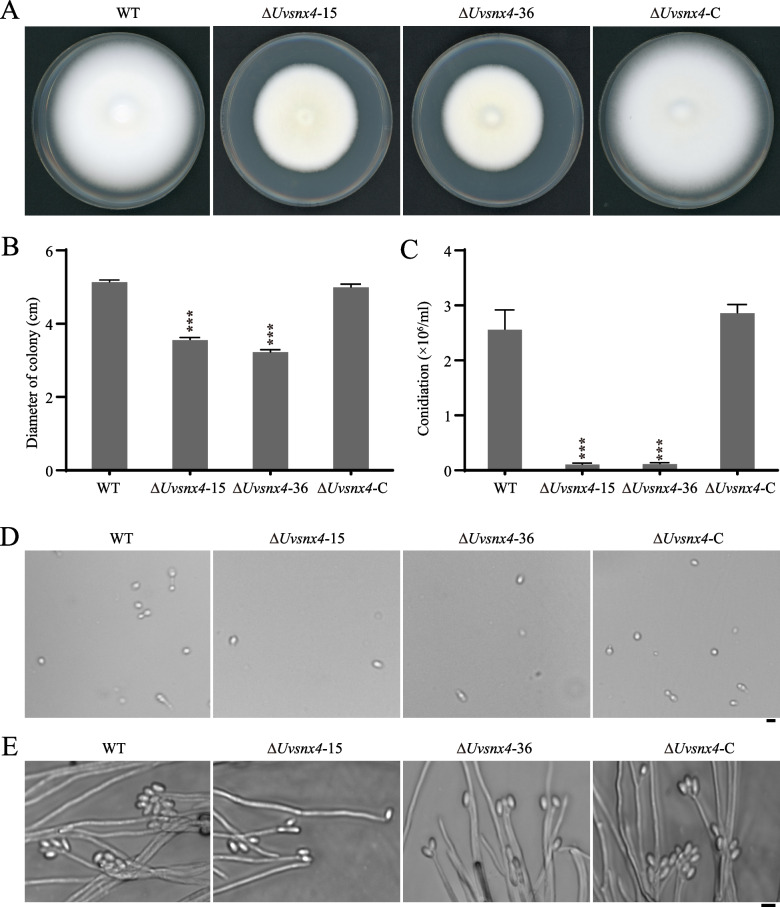
UvSnx4 is required for mycelial growth and conidiation. **A** Disruption of *UvSNX4* reduced mycelial growth in *U. virens*. The indicated *U. virens* strains were cultured on PSA plates for 14 d before being photographed. **B** The colony diameter of Δ*Uvsnx4* is shorter than those of the WT and complemented strains. Diameters of the indicated strains were measured and presented as the mean ± SD from three independent biological repeats. Asterisks represent significant differences at the *p* value < 0.001 level. **C** Disruption of *UvSNX4* led to decreased sporulation. Mycelial plugs of the indicated strains were cultured in PS medium for 7 d to determine the numbers of conidia. **D** Microscopic observation showed fewer conidia in liquid cultures of the Δ*Uvsnx4* mutants. Bar, 5 μm. **E** Less secondary conidia were produced on conidiophores of the Δ*Uvsnx4* mutants. Conidial suspensions were incubated on PSA plates at 28 °C for 3 d before being examined under an Olympus BX53 microscope. Bar, 5 μm

To investigate the roles of UvSnx4 in vegetative growth, the mycelial plugs of the WT, Δ*Uvsnx4*-15, -36, and Δ*Uvsnx4*-C strains were inoculated on PSA (potato sucrose agar) plates for 14 d to measure the colony diameter. The results showed that colony sizes of the ∆*Uvsnx4*-15 and -36 mutants were decreased compared with that of the WT. In contrast, the defects in vegetative growth and colony morphology in Δ*Uvsnx4* were restored in the complementation strain Δ*Uvsnx4*-C (Fig. 2A and B). It was suggested that UvSnx4 is responsible for the vegetative growth of *U. virens*.

The production of spores is the most important factor in *U. virens* infection in rice [2]. In fungi, Snx4 homologues, including MoAtg24, ScSnx4, and FgAtg24, were reported to take part in aerial hyphae and conidiation [19, 23]. To explore the function of *UvSNX4* during spore formation, equal amounts of mycelial plugs from the WT, Δ*Uvsnx4*, and Δ*Uvsnx4*-C cultures were inoculated in liquid PS (potato sucrose) medium at 28 °C and cultured for 7 d. Then, the spores were counted and photographed with a bright field microscope. Compared with the sporulation of the WT and Δ*Uvsnx4*-C strains, the spore production of the Δ*Uvsnx4* strains was reduced to 1/12 of that of the WT and Δ*Uvsnx4*-C strains (Fig. 2C and D), indicating that UvSnx4 is necessary for spore formation in *U. virens*.

To determine the cause of reduced spores in the ∆*Uvsnx4* mutant, we further observed the germination process of spores and the formation of secondary spores. We inoculated spores of the WT, Δ*Uvsnx4*, and Δ*Uvsnx4*-C strains on PSA plates at 28 °C for 3 d. Microscopic observation showed that Δ*Uvsnx4* produced fewer secondary spores than the WT and complemented strains (Fig. 2E). All these data indicated that UvSnx4 is involved in the formation of secondary spores in *U. virens*.

### UvSnx4 is necessary for pathogenesis and stress response in *U. virens*

**Fig. 3:**
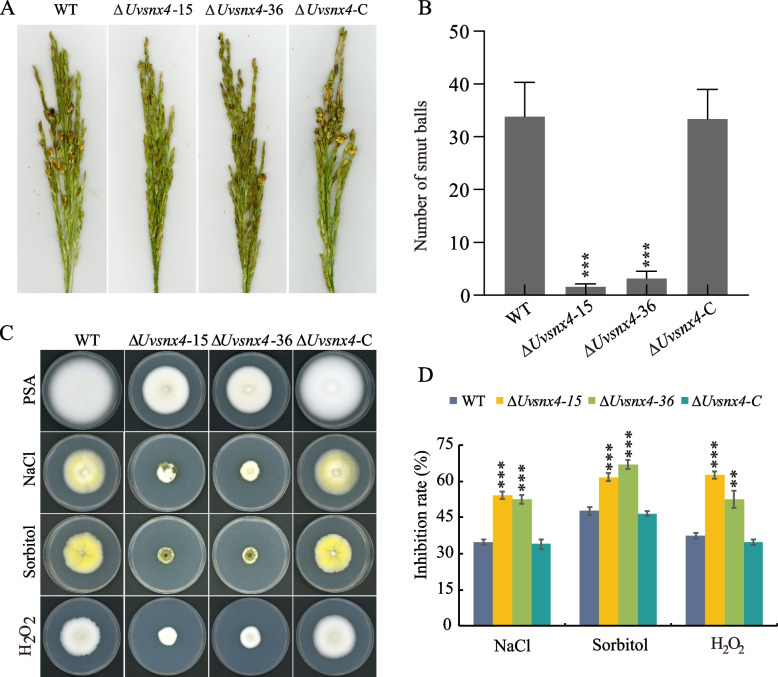
MoSnx4 is required for full virulence and stress response in *U. virens*. **A** Less false smut balls were formed on the panicles inoculated with the Δ*Uvsnx4* mutants. Rice panicles were inoculated with mycelia and spores of the WT, Δ*Uvsnx4*-15, -36, and Δ*Uvsnx4*-C. Diseased panicles were photographed at 21 dpi. **B** Quantification analysis of false smut balls on inoculated rice spikelets. The values are the mean ± SD from three independent experiments, with more than 30 inoculated panicles each time. Asterisks indicate significant differences at *P* < 0.001. **C** The indicated strains were cultured on PSA plates or PSA plates amended with 0.4 M NaCl, 0.7 M sorbitol, or 0.03% H_2_O_2_ at 28 °C for 14 d. **D** Relative inhibition rates of the Δ*Uvsnx4* mutants were higher than those of the WT and complemented strains under osmotic and oxidative stresses. Error bars are standard deviations, and asterisks indicate significant differences at *P* < 0.001 (***) or *P* < 0.005 (**)

During the pathogenic process of *U. virens*, the production of secondary spores contributes to a significant increase in the inoculum of rice plants [24]. Therefore, we speculated that the highly decreased number of secondary spores might weaken infection in the Δ*Uvsnx4* strains. To test this hypothesis, we inoculated panicles of the susceptible rice cultivar Wanxian 98 with mixtures of spores and mycelia of the WT at the booting stage. At 21 dpi, the numbers of rice false smut balls on panicles inoculated with the ∆*Uvsnx4* -15 and 36 strains were fewer than those of the WT and Δ*Uvsnx4*-C strains (Fig. 3A). Detailed statistical analysis indicated that the Δ*Uvsnx4* mutant had significant reductions (8-fold) in the numbers of false smut balls compared with those panicles inoculated with the WT and complemented strains (Fig. 3B). These data suggested that UvSnx4 is involved in fungal pathogenesis in *U. virens*.

Adaptation to different environmental stresses is vital for *U. virens* to accomplish successful infection. Thus, the stress sensitivities of WT, Δ*Uvsnx4*, and the complemented strain Δ*Uvsnx4*-C to osmotic stress and oxidative stress were determined. When cultured on PSA plates amended with 0.5 mol/L NaCl or 0.7 mol/L sorbitol (osmotic stress), the inhibition ratios of *UvSNX4* deletion mutants Δ*Uvsnx4*-15 and Δ*Uvsn4*-36 were significantly higher than that of the WT. The sensitivity of *UvSNX4* deletion mutants to osmotic stresses caused by NaCl and sorbitol could be restored in the complementary mutant Δ*Uvsn4*-C. In addition, the inhibition ratios of Δ*Uvsnx4*-15 and Δ*Uvsn4*-36 to 0.02% H_2_O_2_ (oxidative stress) were also significantly higher than those of the WT and complementary mutant strains (Fig. 3C and D). These results suggested that *UvSNX4* participates in the responses to abiotic stress.

### UvSnx4 is partially localized on mitochondria

**Fig. 4:**
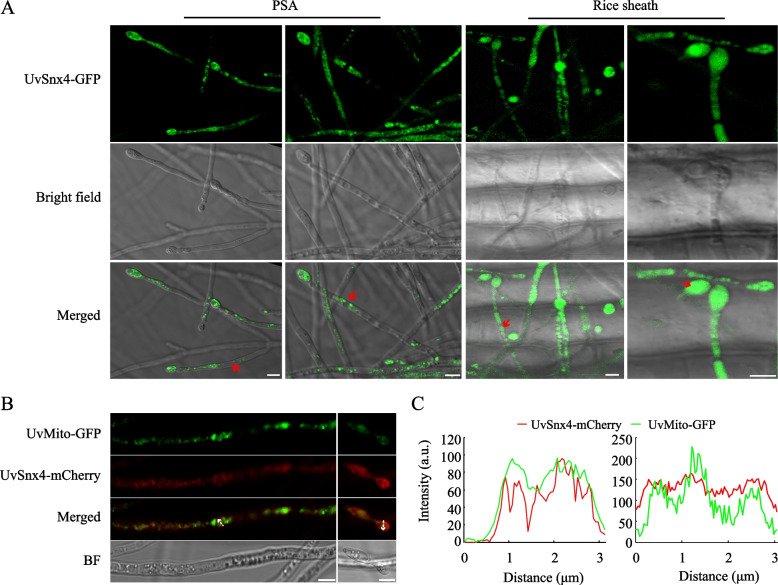
UvSnx4 is partly located on mitochondria. **A** UvSnx4-GFP localized to punctate and tubular structures in the hyphae and spores. The fluorescence of UvSnx4-GFP was visualized by incubating spores of the *UvSNX4-GFP* strain on PSA plates and rice sheathes. Red arrows indicate the vacuoles in the hyphae and spores. Bar, 5 μm. **B** UvSnx4 showed colocalization with the mitochondrial marker UvMito-GFP. Fluorescence microscopic analysis was performed by incubating the *UvSNX4-mCherry*/*UvMito-GFP* strain in PS medium for 2 d. Bar, 5 μm. **C** Line-scan analysis of the fluorescent intensities of UvSnx4-mCherry and UvMito-GFP. The fluorescence intensities of UvSnx4-mCherry and UvMito-GFP in panel B indicated by white arrows were measured with ImageJ software

To examine subcellular localization, the *UvSNX4-GFP* strain was constructed to observe the localization of GFP-tagged UvSnx4. Because UvSnx4 is necessary for spore formation in *U. virens*, we first visualized the localization of UvSnx4 in the hyphae and spores. As shown in Fig. 4A, UvSnx4-GFP localized to punctate and tubular structures, which seem to be mitochondria. In contrast to the distribution of green signals of UvSnx4-GFP in the tubular and punctate structures in the PS medium, part of UvSnx4-GFP was delivered into vacuoles on the rice sheath (Fig. 4A). To further determine whether UvSnx4-GFP colocalized with mitochondria, the fusion expression protein UvSnx4-mCherry was expressed in the *UvMito-GFP* strain, which expresses a mitochondrial DFR1-GFP fusion protein [25]. The resultant *UvSNX4-mCherry*/*UvMito-GFP* strain was used to monitor the subcellular localization of UvSnx4 in mycelia cultured in PS medium. Under laser confocal scanning microscopy, the red fluorescence of UvSnx4-mCherry was dispersed in the cytoplasm of mycelia, and some of the red fluorescence overlapped well with the green fluorescence of UvMito-GFP in the filamentous and punctate structures (Fig. 4B and C). These observations indicated that UvSnx4 partially colocalizes with mitochondria.

### UvSnx4 is required for mitophagy

**Fig. 5:**
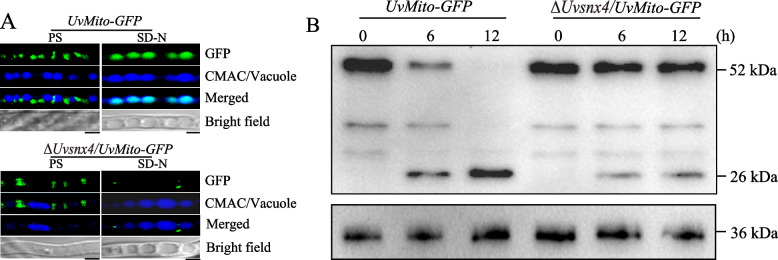
Mitophagy is decreased in Δ*Uvsnx4*. **A** Fluorescence observation revealed that degradation of the mitochondrial marker UvMito-GFP was hindered when *UvSNX4* was deleted. Hyphae of the *UvMito-ATG8* and Δ*Uvsnx4*/*UvMito-ATG8* strains cultured in liquid PS for 2 d were treated with SD-N medium for 6 h. Vacuoles in hyphae were stained with CMAC prior to observation. Bar, 5 μm. **B** Degradation of the fusion protein UvMito-GFP was decreased in the Δ*Uvsnx4*/*UvMito-ATG8* strain. Western blotting was performed with total proteins extracted from mycelia of the *UvMito-ATG8* and Δ*Uvsnx4*/*UvMito-ATG8* strains treated with nitrogen starvation for 0, 6, and 12 h. The GFP levels were detected by anti-GFP antibody with anti-GAPDH antibody as a loading control

In *M. oryzae*, the Snx4 homolog MoAtg24 colocalizes with mitochondria and is required for mitophagy in foot cells [19]. In addition, ScSnx4 is reported to participate in mitophagy induced by nitrogen starvation in *S*. *cerevisiae* [20, 26]. Based on microscopic observation results, we speculated that UvSnx4 may be involved in mitophagy. To test whether UvSnx4 is involved in mitophagy, the *UvMito-GFP* strain was constructed and used to monitor mitophagy in *U. virens*. The mycelia of the *UvMito-GFP* strain were first cultured in liquid PS medium for 3 d and then transferred to nitrogen-starvation SD-N (synthetic dropout medium without nitrogen) medium for 6 h. Vacuoles were stained with CMAC (7-amino-4-chloromenthylcoumarin) dye to observe the degradation of UvMito-GFP. Confocal microscopic observation showed that GFP fluorescence resided in the vacuoles of the nitrogen-starved mycelia, indicating that mitochondrial degradation occurred in vacuoles upon nitrogen starvation (Fig. 5A). In contrast, the GFP signal was rarely captured in the vacuoles of the Δ*Uvsnx4* strain, indicating that UvSnx4 is required for mitophagy (Fig. 5A). In addition to fluorescent observation, the level of mitophagy was monitored by Western blot assay. Consistent with the fluorescence microscopic observation, degradation of the UvMito-GFP fusion protein in the Δ*Uvsnx4* strain displayed a decrease at the indicated time points, suggesting that mitophagy of the Δ*Uvsnx4* strain was defective (Fig. 5B). Overall, these data indicated that UvSnx4 plays an important role in mitophagy in *U. virens*.

### UvSnx4 is involved in nonselective macroautophagy in *U. virens*

**Fig. 6:**
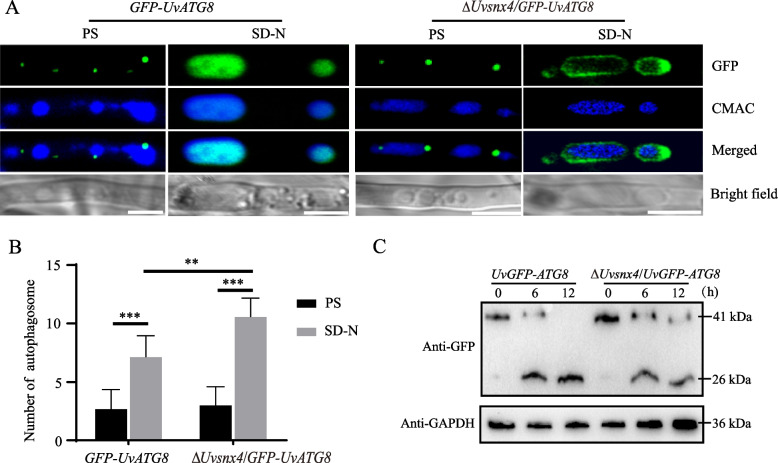
UvSnx4 is involved in autophagy. **A** Delivery of the fluorescent protein UvGFP-Atg8 into vacuoles was impaired in the Δ*Uvsnx4* mutant under autophagy induction conditions. Hyphae of the *UvGFP-ATG8* and Δ*Uvsnx4*/*UvGFP-ATG8* strains cultured in liquid PS for 2 d were treated with SD-N medium for 6 h. Vacuoles in hyphae were stained with CMAC staining prior to observation under confocal microscopy. Bar, 5 μm. **B** The number of autophagosomes increased in the Δ*Uvsnx4* mutant under nitrogen starvation conditions. More than 20 hyphal segments were observed to count the fluorescent dots indicative of autophagosomes. The asterisks indicate a significant difference between the WT and Δ*Uvsnx4* strains (**, *P* < 0.005). **C** Deletion of *UvSNX4* resulted in defects in UvGFP-Atg8 degradation. Total proteins were extracted from mycelia of the *UvGFP-ATG8* and Δ*Uvsnx4*/*UvGFP-ATG8* strains treated with SD-N medium for 0, 6 and 12 h. The GFP levels were detected by western blotting with anti-GFP antibody. The GAPDH protein was used as a loading control and detected with an anti-GAPDH antibody

Mitophagy is a cellular process that selectively degrades dysfunctional mitochondria via autophagy [15]. Given that Snx4 plays an important role in mitophagy in fungi [18, 19, 21, 26], we wanted to explore whether UvSnx4 also participates in macroautophagy. To monitor macroautophagy under starvation conditions, *UvSNX4* was deleted in the *GFP-UvATG8* strain [11]. Upon treatment with nitrogen starvation, GFP fluorescence in the *GFP-UvATG8* strain accumulated in CMAC-dyed vacuoles. In contrast, the GFP signal was rarely detected in vacuoles of the Δ*Uvsnx4*/*GFP-UvATG8* strain under the induced condition, indicating that UvSnx4 participates in macroautophagy in *U. virens* (Fig. 6A). Furthermore, the numbers of autophagosomes were induced in both *GFP-UvATG8* and Δ*Uvsnx4*/*GFP-UvATG8* strains upon nitrogen starvation. However, the number of autophagosomes in the Δ*Uvsnx4*/*GFP-UvATG8* strain was significantly lower than that in the *GFP-UvATG8* strain upon nitrogen starvation induction (Fig. 6B). Consistent with microscopic observation, western blotting analysis showed that degradation of the GFP-UvAtg8 fusion protein was reduced in the Δ*Uvsnx4*/*GFP-UvATG8* strain compared with that in the *GFP-UvATG8* strain upon nitrogen starvation (Fig. 6C), indicating that disruption of *UvSNX4* resulted in defects in autophagy. All these data suggested that in addition to mitophagy, UvSnx4 is also involved in autophagy in *U. virens*.

In *F. graminearum*, FgAtg24, a homolog of UvSnx4, was found to interact with the autophagy-related proteins FgAtg1, FgAtg11, FgAtg17, and FgAtg20. To determine whether these interactions are conserved, yeast two-hybridization assays were conducted. As shown in Figure S3, transformants expressing UvSnx4BD and UvAtg11AD, UvSnx4BD and UvAtg17, and UvSnx4BD and UvAtg20AD could grow on SD-Leu/Trp/His/Ade plates, indicating that UvSnx4 has conserved interactions with UvAtg11, UvAtg17, and UvAtg20. In contrast, UvSnx4 did not interact with UvAtg1. Therefore, we postulated that UvSnx4 might participate in mitophagy and autophagy by interacting with autophagy-related proteins.

## Discussion

Sorting nexins are important proteins with conserved functions that play crucial roles in regulating protein intracellular trafficking, such as endocytosis, endosomal sorting, and signaling. In this study, we characterized the sorting nexins UvSnx4 in *U. virens* and found that it is required for mycelial growth, sporulation, stress adaptation, and pathogenicity. Furthermore, we revealed an important linkage between UvSnx4 and the regulation of mitophagy and autophagy in this fungal pathogen.

Snx4 homolog-mediated biological functions are similar in different filamentous fungi, but the regulatory mechanisms seem to vary. In *M. oryzae*, the mitophagy auxiliary gene *MoATG24*, a sorting nexin related to yeast Snx4, is involved in conidiation and pathogenicity [19]. In the wheat scab fungus *F. graminearum*, FgAtg24, a homolog of Snx4, is also necessary for growth, conidiation, and pathogenicity [23]. In *Cochliobolus heterostrophus*, sorting nexin Snx4 (Atg24) interacts with Snx41 (Atg20) and participates in fungal growth, asexual development and virulence [27]. Consistently, in this study, we revealed that ∆Uvsnx4 showed a defect in growth, conidiation and pathogenicity, indicating that Snx4 homologues are conserved in regulating growth, conidiation, and pathogenicity. Interestingly, the molecular mechanisms underlying which Snx4 homologues participate in regulating these biological processes are not the same. Deletion of MoAtg24 interrupts mitophagy in foot cells and invasive hyphae, leading to decreased conidiation and invasive growth. However, MoAtg24 is not required for pexophagy and macroautophagy [19]. In contrast, in *F. graminearum*, FgAtg20 interacts with FgSnx4/FgAtg24, participating in the cytoplasm-to-vacuole targeting pathway, macroautophagy and pexophagy, but not mitophagy. Moreover, direct interactions have been found between FgSnx4 and FgAtg1, FgAtg11, FgAtg17, and FgAtg20 [28]. In our study, we found that UvSnx4 is involved in mitophagy and macroautophagy, which are important for conidiation and pathogenicity in *U. virens* [5, 29]. In comparison with FgSnx4, UvSnx4 interacts with UvAtg11, UvAtg20 and UvAtg17 but not with UvAtg1. In the future, it is necessary to further investigate the difference and similarity of UvSnx4 in other types of autophagy, such as pexophagy and the CVT pathway in *U. virens*.

In this work, we assumed that defects of Δ*Uvsnx4* in pathogenicity are associated with impaired autophagy and mitophagy in *U. virens*. Autophagy is important for development, stress adaptation and pathogenicity in different fungal pathogens [14]. In *U. virens*, our previous study revealed that the core autophagy protein UvAtg8 mediates the stress response, fungal conidiation, and pathogenicity [11]. Moreover, another autophagy-related gene, UvAtg7, is also reported to be required for cell stress responses, asexual reproduction and virulence, further confirming the crucial roles of autophagy in the development and pathogenicity of *U. virens* [5]. Here, our results suggested that deletion of *UvSNX4* led to defects in macroautophagy-mediated degradation, as confirmed by the reduced degradation of GFP-UvAtg8. In addition to macroautophagy, another role of Snx4 in pathogenicity may be mitophagy. Although mitophagy has not been depicted in *U. virens*, it has been reported to regulate conidial differentiation and pathogenicity in several fungi, including *M. oryzae* and *Aspergillus. oryzae. B. bassiana*, and *Colletotrichum higginsianum* [19, 30, 31]. In this study, we found that UvSnx4 is required for mitophagy, which may influence the formation of secondary conidia and lead to infection defects. The fluorescence of UvMito-GFP could be transported into vacuoles during sporulation on the rice surface, implicating the occurrence of mitophagy during the infection process. In addition, two UvSnx4-mediated biological processes, mitophagy and macroautophagy, may be related. Yeast two-hybrid analysis showed that UvSnx4 interacts with both core autophagy proteins UvAtg17 and selective autophagy proteins UvAtg20 and UvAtg11. Recently, it was reported that Snx4 assists in targeting the transcription factor Ssn2 for vacuole degradation and regulating *ATG* gene expression. The sorting nexin heterodimer Snx4-Atg20 binds Atg17, contributing to substrate transfer. Therefore, we postulated that UvSnx4 might control development and pathogenicity by regulating mitophagy and macroautophagy in *U. virens*.

In addition to critical roles in mitophagy and macroautophagy, UvSnx4 plays crucial roles in the stress response, including osmotic stress and oxidative stress. In filamentous fungi, the MAPK Hog1-mediated signaling pathway is responsible for these two kinds of stresses [32, 33]. In contrast, Hog1 in *U. virens* is required for hyperosmotic stress but not for oxidative stress [34]. An adaptor protein UvSte50, interacting with UvHog1, participates in responding to osmotic stress and oxidative stress [35]. In *M. oryzae*, MoAtg24 is also confirmed to be required for the oxidative stress response [19]. Whether UvSnx4 has crosstalk with the UvHog1-mediated signaling pathway needs to be investigated in the future.

This study provides insight into the biological roles of UvSnx4 in the development and pathogenicity of *U. virens* and reveals its vital regulatory roles in mitophagy and macroautophagy. These results not only expand our understanding of the mitophagy and autophagy regulatory mechanisms in fungi but also provide instructional knowledge for the management of rice false smut disease.

## Materials and methods

### Sequence analysis

The gene and protein sequences of *SNX4* homologues used in this study were obtained from the National Center for Biotechnology Information (NCBI, https://www.ncbi.nlm.nih.gov/) database. The motifs of UvSnx4 and other homologues were scanned by the software tool SMART (http://smart.embl-heidelberg.de/), and the phylogenetic tree of Snx4 homologues was constructed using MEGA 7.0 with a neighbor-joining algorithm [36].

### Strains and growth conditions

The *U. virens* strain HWD-2 kindly provided by Prof. Junbin Huang of Huazhong Agriculture University (China) was used as the wild-type (WT) strain in this study. The *U. virens* strains used in this study were cultured on PSA (potato 200 g/L, sucrose 20 g/L, and agar 20 g/L) plates in a dark chamber at 28 °C. To determine the phenotypic change in vegetative growth, mycelial plugs were cultured on PSA plates for 14 d and then photographed. To harvest mycelia, mycelial plugs were inoculated in liquid PS (potato 200 g/L and sucrose 20 g/L) medium for 7 d before collection. Then, conidia were collected by filtration of cultures to determine the conidial concentrations [11]. The conidial germination assay was conducted by spraying the conidial suspension on the PSA plate surface and the rice sheath for 3 d.

### Construction of plasmids and transformation

To generate the vector expressing *UvMito-GFP*, approximately 1.5 kb of the 5’ UTR and coding regions of *UvDFR1* (*UV8b_7457*, dihydrofolate reductase) were cloned and then ligated to the *pFGL822-GFP-TrpC* terminator [37]. The resultant plasmid was sequenced and subsequently transformed into the WT by *A. tumefaciens-*mediated transformation (ATMT). The resultant transformants were confirmed by PCR and epifluorescence microscopic observation.

To obtain the null mutants of *UvSNX4*, a gene deletion strategy was adopted as previously reported [38]. Briefly, approximately 1 kb of the 5’ and 3’ flanking sequences of *UvSNX4* were cloned with primers and ligated to the flanking sites of the *hygromycin phosphotransferase gene cassette* in pFGL821 (Addgene, 58,223). The resultant construct was sequenced and then introduced into the HWD-2, *GFP-UvATG8*, and *UvMito-GFP* strains by ATMT to obtain the deletion mutants Δ*Uvsnx4*, Δ*Uvsnx4*/*GFP-ATG8*, and Δ*Uvsnx4*/*UvMito-GFP*, respectively. To construct complementation strains, the full-length genomic copy composed of 1.6 kb of the promotor and coding region of *UvSNX4* was cloned and then inserted into pFGL823 [29]. The sequenced construct was transformed into Δ*Uvsnx4* by ATMT. The mutants used in this study were verified by PCR, Southern blot assay, and qRT‒PCR as previously described (primers used in amplification are listed in Table S1) [39].

### Stress sensitivity assay

To test the sensitivities of *U. virens* strains to different abiotic stress conditions, the mycelial plugs of the WT, ∆*Uvsnx4* mutants, and complementation strain ∆*Uvsnx4*-C were cultured on PSA plates supplemented with 0.4 M NaCl, 0.7 M sorbitol or 0.03% H_2_O_2_ at 28 °C for 14 d. Pictures were taken to display the vegetative growth of the indicated strains under various stress conditions. The calculation of the relative growth inhibition rate was written as follows: relative growth inhibition rate = (average diameters of strains cultured on the PSA plate – average of strains cultured on the PSA plate amended with various stress regents)/average of the strains cultured on the PSA plate × 100%. All of the growth experiments were repeated three times with three replicates each time.

### Inoculation assay

The infection assay was performed as previously reported [11]. *U. virens* strains were first cultured in 50 mL of PS medium for 7 d. The cultures containing mycelial balls and spores (1 × 10^6^ spore/mL) were broken down. Then, 2 mL of the resultant strain mixture was inoculated into the panicles of rice plants (*Oryza sativa* L., cultivar Wanxian98) at the booting stage. The inoculated rice plants were treated at 22 °C in the dark and then transferred to conditions of 25 °C and 90% humidity. The numbers of false smut balls on each panicle were counted and recorded at 21 d post inoculation (dpi).

### Observation of UvSnx4 subcellular localization

To determine the subcellular location of UvSnx4, the UvSnx4-mCherry fusion expression vector was constructed. The genomic sequence containing the native promoter and coding region of *UvSNX4* were amplified with primers and ligated into the vector *pFGL823-mCherry-TrpC* terminator. The resultant *UvSNX4*-mCherry plasmid was transformed into the *UvMito-GFP* and *GFP-UvATG8* strains by ATMT. Similarly, the *pFGL822-UvSNX4-GFP-TrpC* construct expressing UvSnx4-GFP was constructed and then transformed into the WT strain.

### Induction and detection of mitophagy and autophagy

To detect mitophagy and autophagy, the *UvMito-GFP* and *GFP-UvATG8* strains were first cultured in 100 mL of PS medium for 3 d and then transferred to synthetic dropout medium without nitrogen (SD-N, yeast nitrogen base without amino acids 1.7 g/L, glucose 20 g/L) for 0, 6, and 12 h [11, 19]. Vacuoles in mycelia were stained with 10 μM CMAC dye (7-amino-4-chloromethylcoumarin, Molecular Probes, C2110) for 30 min before microscopic observation. To detect the degradation of the mitophagy and autophagy marker proteins UvMito-GFP and GFP-UvAtg8, total proteins were extracted from the indicated mycelial powders ground with liquid nitrogen. Total protein powders were dissolved in lysis buffer (30 mM Tris–HCl pH 7.4, 1 mM EDTA, 100 mM NaCl, 1% Triton 100) supplemented with proteinase inhibitor. Equal amounts of protein samples (50 μg) were separated with 12% SDS‒PAGE and then transferred to a PVDF (polyvinylidene fluoride) membrane. The PVDF membrane was incubated with the primary antibodies anti-GFP (Cat. No. ET1607-31, HuaBio, China) or anti-GAPDH (Cat. No. R1208-3, HuaBio, China). The amount of GAPDH protein was used as a loading control. The results were detected with an ECL chemiluminescent kit (GS-710, Bio-Rad, USA).

### Fluorescence microscopy

To observe the fluorescent localization of UvSnx4, UvMito-GFP, and UvGFP-Atg8, mycelia and conidia were collected and stained with CMAC. The fluorescent signals were captured with an inverted confocal microscope (Zeiss LSM 700) with a Plan-Apochromat 63 (NA 1.40) oil immersion lens. The fluorescent signals of GFP, CMAC, and mCherry were excited at 488 nm (Em. 505–530 nm), 405 nm (Em. 430–470 nm), and 555 nm (Em. 600–625 nm), respectively. The fluorescent images were organized with the software Adobe Illustrator CS6 and ImageJ.

## Supplementary Information

**Additional file 1: Figure S1.** Alignment of Snx4 homologues. **Figure S2.** Verification of *UvSNX4* null mutants. **Figure S3.** UvSnx4 interacts with UvAtg11, UvAtg17, and UvAtg20, as determined by yeast two-hybrid assays. **Table S1.** Primers used in this study.

## Data Availability

The data that support the findings of this study are available upon reasonable request.
