# Supplementary material for: UvSnx4 is required for conidiation, pathogenicity and stress responses by regulating mitophagy and macroautophagy in Ustilaginoidea virens
Source: Crop Health. 2023 Aug 10;1(1):2. doi: 10.1007/s44297-023-00002-z (PMC12825972; doi:10.1007/s44297-023-00002-z)
Supplement: Supplementary file 1 — Additional file 1: Figure S1. Alignment of Snx4 homologues. Figure S2. Verification of UvSNX4 null mutants. Figure S3. UvSnx4 interacts with UvAtg11, UvAtg17, and UvAtg20, as determined by yeast two-hybrid assays. Table S1. Primers used in this study. [file 44297_2023_2_MOESM1_ESM.docx]

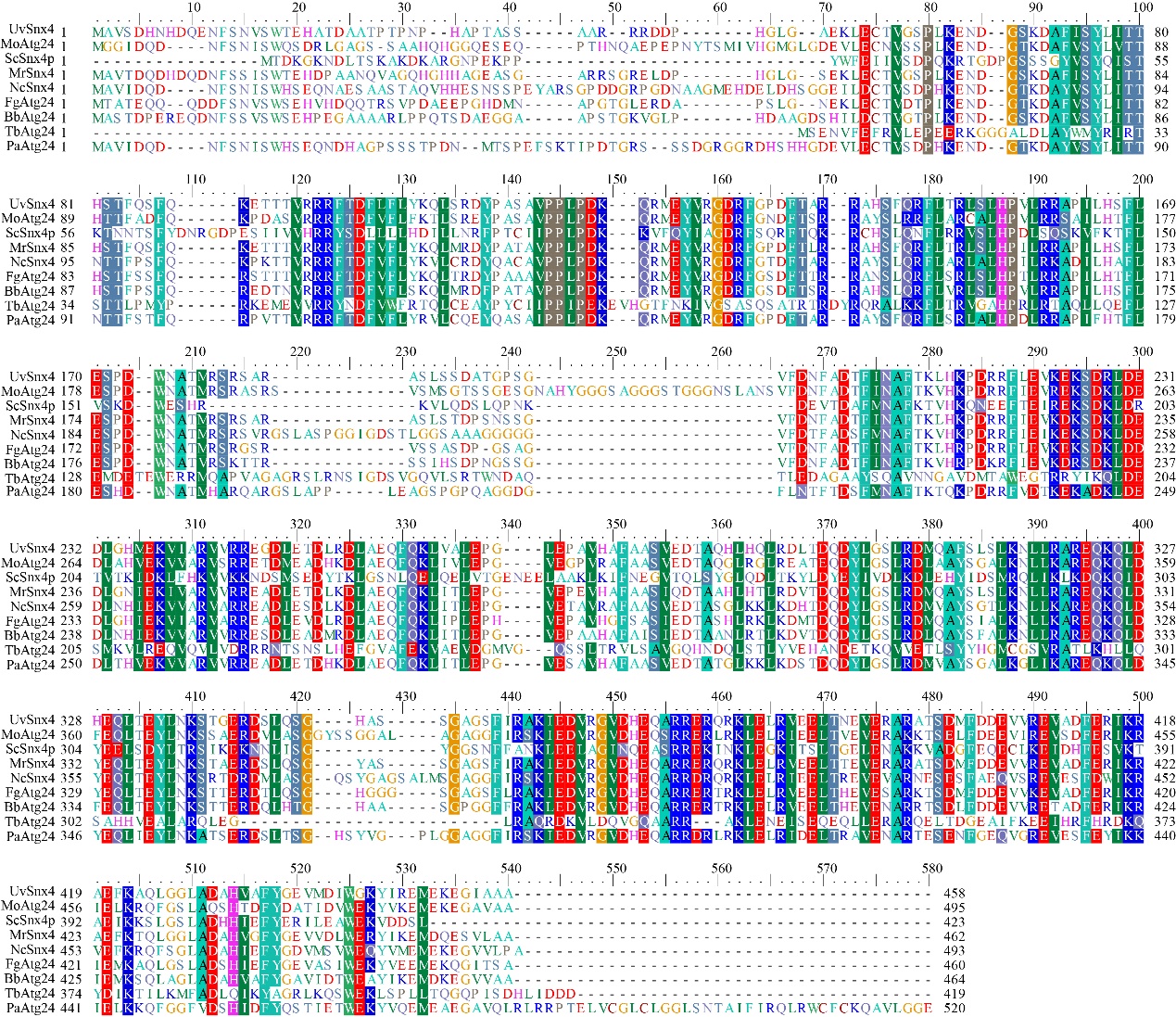


Figure S1. The alignment of Snx4 homologs. Amino acid sequences of nine Snx4 homologs were aligned with the MEGA software. The identical amino acids were labelled with different colors.


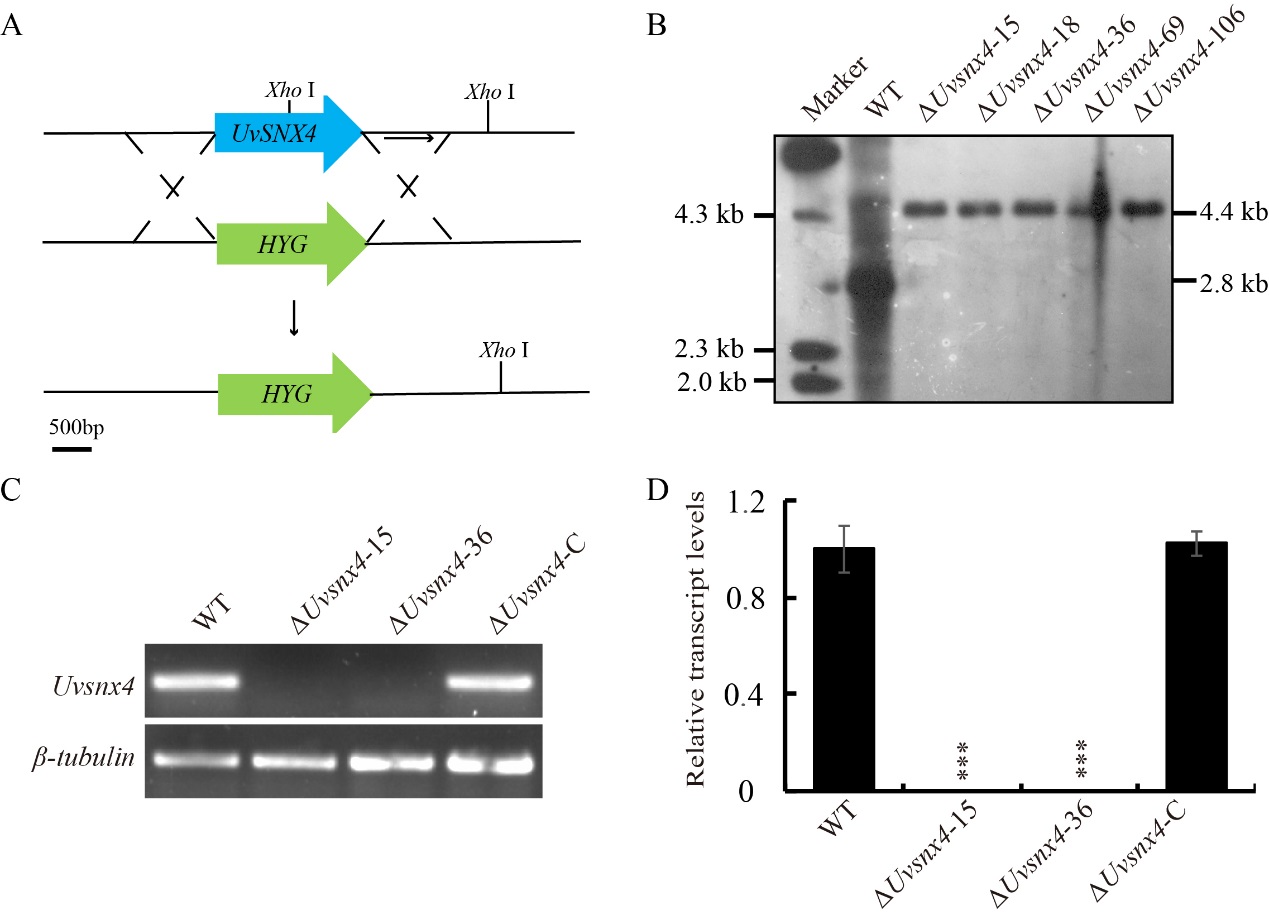


Figure S2. Verification of *UvSNX4* null mutants. (A) Schematic of gene knockout strategy. The knockout vector of *UvSNX4* was constructed by ligating the flanking sequences of *UvSNX4* into flanking regions of *hygromycin phosphotransferase gene cassette* (*HYG*). (B) Southern blot analysis of the Δ*Uvsnx4* mutants. Genomic DNA isolated from the WT and Δ*Uvsnx4* mutants was digested with *Xho*I. The WT strain showed a 2.8 kb-band, and null mutants Δ*Uvsnx4*-15, -18, -36, -69, and -106 exhibited the predicted 4.4 kb-band. (C) RT-PCR analysis of the Δ*Uvsnx4* mutants. (D) Quantitative PCR analysis of *UvSNX4* in the WT and Δ*Uvsnx4* mutants. Gene expression levels of *UvSNX4* were determined by qPCR. The results were calculated from three biological repeats. Asterisks indicated significant difference at *p* value < 0.001 level.


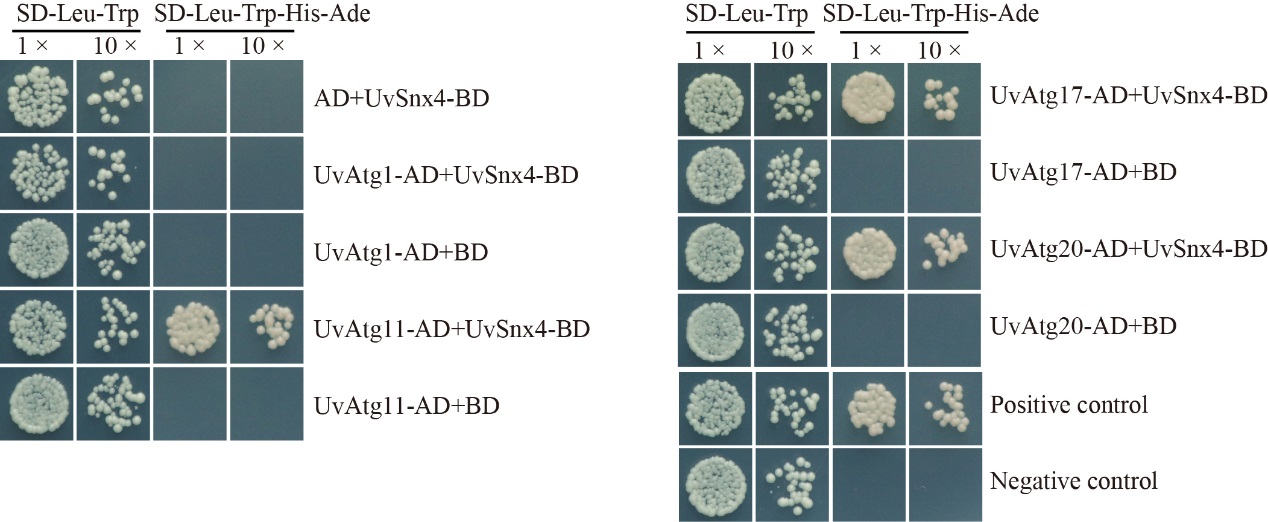


Figure S3 UvSnx4 interacts with UvAtg11, UvAtg17, and UvAtg20 determined by yeast-two-hybridization assays. UvSnx4 was ligated into pGBKT7 vector and used as a bait. UvAtg1, UvAtg11, UvAtg17, and UvAtg20 were ligated into pGADT7 as preys. Yeast transformants expressing a pair of bait and prey were diluted on SD-Leu/Trp/His/Ade plates and cultured for 3 d at 30 °C.

| **Primer** | **Sequence (5’-3’)** | **Application** |
| --- | --- | --- |
| UvSNX4-5F | cgagaattcgagctcggtaccAACGAAGAGAGCCAGAGCACG | Amplifying 5’ flank sequence of *UvSNX4* for gene deletion |
| UvSNX4-5R | ttcaatatactcgactctagaGTTCCGACGCTCAGAGGAAA |  |
| UvSNX4-3F | ccggaaccagtcgacctgcagTTGTGCTCTTGTTGGCGTTG | Amplifying 3’ flank sequence of *UvSNX4* for gene deletion |
| UvSNX4-3R | ttgagtacccaattcaagcttTCGACAAGTTCCCCACGTTT |  |
| UvSNX4-TF | CCCCTCGACCCTGCTTGTAC | Transformants screening |
| p821-5R | ACCTCCACTAGCTCCAGCCAAG |  |
| UvSNX4-cf | tatggagaaactcgagaattcCAAGGAAACAGGCTTGCCAC | Construction of complementation vector |
| UvSNX4-cr | ctagtggatcccccgggtaccCTGGTCTGGCTGGACTTGTG |  |
| UvSNX4-GFPF | tatggagaaactcgagaattcCAAGGAAACAGGCTTGCCAC | Construction of fluorescent localization vector |
| UvSNX4-GFPR | ctcgcccttgctcacggtaccGGCGGCGGCTATGCCCTC |  |
| UvSNX4-mCherryF | AGAAACTCGAGAATTCCAAGGAAACAGGCTTGCCAC |  |
| UvSNX4- mCherryR | CCTTGCTCACGGTACCGGCGGCGGCTATGCCCTC |  |
| UvMito-GFPF | TATGGAGAAACTCGAGAATTCAAGAACGCCTATCCAACGG |  |
| UvMito-GFPR | CTCGCCCTTGCTCACGGTACCTGCCCGCTTCACGGGTTTCT |  |
| UvSNX4RT-F | ACTTTGCCGACACCTTCATC | Analysis the expression level of *UvSNX4* gene of U. virens |
| UvSNX4RT-R | GGCAATCACCTTTTCCATGTG |  |
| UvACTIN-F | CCATGTACCCTGGTCTCTCC | Analysis the expression level of *ACTIN* gene of *U. virens* |
| UvACTIN-R | CTCTCGTCGTACTCCTGCTT |  |
| UvSNX4-probeF | TTGTGCTCTTGTTGGCGTTG | Amplification of probe for Southern blot assay |
| UvSNX4-probeR | TCGACAAGTTCCCCACGTTT |  |

Table S1. Primers used in this study.
